# The effect of organizational differentiation in football training on young football players

**DOI:** 10.3389/fpsyg.2025.1565594

**Published:** 2025-11-11

**Authors:** Olav Størdal, Terje Dalen, Pål Lagestad

**Affiliations:** Department of Teacher Education and Arts, Nord University, Levanger, Norway

**Keywords:** soccer, differentiation, adolescents, psychology, physiology

## Abstract

**Introduction:**

The Norwegian Football Federation (NFF) has a primary goal of providing children and adolescents with a good football offer and positive football experiences, where differentiation is one way to achieve this. The aim of the present study was to investigate the effect organizational differentiation has on psychological and physical variables in 13–14-year-old male football players.

**Methods:**

An intervention study with an experimental randomized crossover design was used, where players from three football teams participated in two differentiated training sessions and two non-differentiated training sessions. The four training sessions had the same exercises, with the same coach. The physical measurements involved heart rate, number of accelerations, number of sprints and total distance covered measured by Polar Team Pro GPS, while the psychological variables measured in a questionnaire was wellbeing, mastery, joy, satisfaction and experienced development. Wilcoxon nonparametric tests were used to compare the results from differentiated and non-differentiated sessions.

**Results:**

The results showed that players with upper-level skills (UG) experienced significantly higher enjoyment, satisfaction, and development in differentiated training compared to non-differentiated training and preferred differentiated training. No significant differences were observed in psychological variables between differentiated and non-differentiated training among lower-group players. Furthermore, all players had more accelerations and increased their total distance covered during differentiated training compared to non-differentiated training regardless of group. Finally, players in UG had a higher average heart rate during the entire session, as well as in the sub-exercises SSG, 2v2+1 games, and rondo 4v1.

**Discussion:**

The results suggest that organizational differentiation positively affects young players' physical variables, especially players with upper-level skills. Organizational differentiation also positively affects psychological variables of young players with upper-level skills. However, careful consideration should be given to the potential long-term consequences of differentiation for lower-skilled players, especially regarding social belonging and self-perception.

## Introduction

The Norwegian Football Federation (NFF) states that the main goal of children's and youth football (soccer) is to provide as many children and young people as possible with good football opportunities and experiences, where a differentiated training offer is one of the ways we achieve this (NFF, 2021a). Differentiation provides good football opportunities and experiences by facilitating a mastery-oriented climate and mastery for each player (Csikszentmihaly, 1975, 1990). Furthermore, research has shown that how football training is organized have an effect on enjoyment, motivation, long-term participation, and personal development, and should be organized with principles that support intrinsic motivation and positive psychological outcomes (Erikstad et al., 2021).

Differentiation is highlighted as a key mechanism in the training of talented sportsmen (Bon, 2009). According to NFF (2021a) differentiation can occur through various conditions, different learning moments, different exercises, different execution requirements, division by skill level in parts of the session, or training groups by level or training enthusiasm. The advantage of differentiating by level is that progress and enjoyment are created by meeting even competition (Berntsen and Johansen, 2020). Differentiating by level is achieved when the group is organizationally differentiated, and one does not face too hard or too weak opposition. Facing opponents of similar skill level during training enhances the likelihood of success by allowing athletes to perform more effective actions and build mastery through repeated positive experiences. Ward et al. (2019) examined how grouping youths by skill level according to ball related games affected performance and physical activity levels. The participants were placed in high skill groups and low skills groups, but also in mixed skill level groups. The results showed that placing youths in high skill groups and low skills groups led to increased opportunities for success and higher levels of moderate-to-vigorous physical activity. Furthermore, the study of Weiss (2020) examined how grouping by skill level (e.g., high vs. low skill) influenced youth through several psychological mechanisms such as perceived competence, self-confidence, motivation and relatedness. The study of Ward et al. (2019) and Weiss (2020) provides a strong empirical basis for defining organizational differentiation as: *the practice of grouping students into groups based on assessed skill levels, with the aim of optimizing performance, and learning outcomes during physical activity*.

Csikszentmihaly's (1975) flow theory promotes a balance between challenge and skills, which can occur by differentiating by skill level or physical development. Thus, enjoyment and happiness can come because of mastery and social belonging (Csikszentmihaly, 1975). Entering the flow state positively affects athletes' intrinsic motivation (Csikszentmihaly, 1975). This state occurs when there's a balance between challenge and skills, leading to absorption in the activity and joy in participation (Csikszentmihaly, 1975, 1990). The flow state arises from intense activities requiring high focus, often due to appropriately challenging resistance (Biasutti, 2011). Low-intensity activities don't necessitate focus, preventing entry into the flow state. This state also occurs when activities are challenging and involve risk (Biasutti, 2011). In sports, flow theory aims to provide athletes with optimal experiences (Csikszentmihaly, 1990). For young athletes, entering the flow state is particularly beneficial for development, as mastery, wellbeing, and joy foster growth. As skills develop, challenges must increase to maintain this state; otherwise, athletes may experience boredom if training is no longer challenging (Hood, 2007). Literature searches indicate that no study directly and empirically applies Csikszentmihaly's flow theory specifically to youth football training. However, a study of Norsworthy et al. (2023) showed that Csikszentmihaly's flow theory was related to positive outcomes in youths regarding performance, competence, wellbeing, intrinsic motivation, stress and anxiety. Researchers suggest that game sequences should be tailored to provide appropriate challenges for all participants (Johansen, 2019; Berntsen and Johansen, 2020).

The Norwegian Sports Federation's (NFF, 2021b) policy emphasizes the vision of “joy of sports for all,” highlighting the importance of mastery and wellbeing to prevent dropout (Seipel, 2005). Differentiation, or adapting programs to various skill levels, can promote mastery, joy, wellbeing, and development (Berntsen and Johansen, 2020; Csikszentmihaly, 1975, 1990). This approach aligns with NFF's vision of “as many as possible, for as long as possible, as good as possible” (NFF, 2021b). Differentiation leads to individualized training, providing appropriate challenges for everyone (Csikszentmihaly, 1990). While individualization is challenging in team sports, it can be achieved within the training group framework (Ronglan, 2018; Hallèn and Ronglan, 2022). Organizational differentiation can affect players differently, and game sequences should be tailored to provide challenges according to each player's level (Johansen, 2019; Berntsen and Johansen, 2020).

In football, players need a high level of both aerobic and anaerobic capacity to succeed. At ages 13–14, players are in different phases of pubertal development, leading to significant differences in anthropometry, technical skills, and physical abilities like VO_2max_, strength, and sprint speed (Naisidou et al., 2017). These disparities make differentiation crucial at this age. Differentiation may affect the physical and psychological stress on players, though studies on its impact in football are rather scarce. Some research indicated that differentiated physical education programs improve physical fitness (Sitovskyi et al., 2019; Savliuk et al., 2020; Nagovitsyn et al., 2021). Bolotin and Bakayev (2017) rank differentiation and individualization as key tools for enhancing speed and explosive strength in young football players, stressing the need for training tailored to each athlete's level and background. Sitovskyi et al. (2019) found that differentiation improved physical performance. However, Bon (2009) and Bolotin and Bakayev (2017) noted that differentiation can also impact mental and emotional factors. The Norwegian Directorate for Education and Training (2023) highlighted the importance of social belonging in differentiation, particularly in schools. Bon (2009) mentioned that being placed in the “weakest group” can have mixed effects, emphasizing the need for variation and a focus on mastery over performance. Based on the above findings, the aim of the present study was to investigate the effect organizational differentiation related to skill level has on psychological (perceived mastery, wellbeing, satisfaction, joy, and development) and physical (total distance, number of sprints, number of accelerations, and average heart rate) variables in 13–14-year-old male football players. The hypothesis was that organizational differentiation in high and low skill level would have a positive effect on the psychological and physical variables included. The results will be discussed according to previous research and considering Csikszentmihaly's (1975) flow theory.

## Method

To investigate the effect of organizational differentiation on psychological and physical variables, an intervention study with a counterbalanced crossover design was used. The intervention consisted of conducting the same session plan four times over 2 weeks. Randomization was performed at the team level, with three teams randomly assigned to begin with either the differentiated or non-differentiated condition. Each team experienced both conditions: two training sessions with differentiation and two without. Counterbalancing was applied to control for order effects, with conditions implemented on separate days to allow for recovery and psychological reset (see Table 1). A wash-out period was not included between conditions, as the intervention consisted of isolated training sessions with expected short-term effects and no long-term physiological effects or psychological carryover. Furthermore, outcome measures were collected immediately after each session, minimizing the risk of residual effects influencing subsequent conditions. Each condition was implemented on separate days across 2 weeks, allowing for natural recovery and psychological reset between sessions. The within-subject design allowed each participant to serve as their own control, increasing sensitivity to detect intervention effects. Using a crossover design with counterbalancing is a recognized strategy to minimize carry-over effects (Gondaliya and Divecha, 2022).

**Table 1:** Differentiated and non-differentiated training among the three teams.

**Team number**	**First week**	**Second week**
Team 1	Differentiated	Non differentiated
Team 2	Non differentiated	Differentiated
Team 3	Non differentiated	Differentiated

The research project was approved by the Norwegian data protection agency (SIKT), and the information consent form was approved and signed by all players and guardians before the first training session (SIKT, reference code 543176). Information was provided about the collection of questionnaire data and physical data, but no information was given about the research question to avoid influencing the effort during the training sessions.

### Participants

Using a stratified sample, five clubs with Boys U14 teams with more than 15 players were contacted. One team did not respond, one team declined, and three teams consisting of 13–14-year-old boys agreed to participate, resulting in a total of 71 participants. The exclusion of female players was due to practical access to teams and the need to ensure sample homogeneity. The players were differentiated into two groups based on skill level, upper-level group (UG) and lower-level group (LG), conducted by the head coaches of the respective teams. The coaches were instructed to differentiate the players based on a holistic assessment of their football skills, including technical, tactical, and physical abilities, with particular emphasis on technical skills as the most important criterion. Injuries, sickness, and other circumstances prevented some participants from taking part in data collection during both the differentiated and non-differentiated training sessions. Because of this, a total of 30 players in UG and 20 players in LG had valid physical data, while 28 players in UG and 30 players in LG had valid psychological data (see Table 2).

**Table 2:** Number of players in upper-level group (UG) and lower-level group (LG) with valid data for the different teams, as well as the number of players per training session.

**Team number**	**Valid physical data UG**	**Valid physical data LG**	**Valid psychological data UG**	**Valid psychological data LG**	**Players at the training sessions**
Team 1	7	6	8	8	16–18
Team 2	10	5	9	6	16–31
Team 3	13	9	11	16	30–50
Total	30	20	28	30	

### Description of the intervention

Data collection took place in October and November for teams 1 and 2, while it was conducted in April for team 3. All training sessions, both with and without differentiation, were conducted on the artificial turf fields of the respective teams at their normal training times. In this way, the two non-differentiated training sessions took place at the same location and time as the differentiated training sessions for all three teams. The training session and exercises was organized as a typical and traditional football training for this age group based on the recommendations of NFF, and the same coach conducted all the training sessions (Appendix 1). All teams started the training by performing part of the warm-up exercise “FIFA 11+” followed by a technical circle for a more specific warm-up. After that the players performed a rondo 4vs1-exercise in squares of 5x5 meters. The rondo exercise was followed by a passing exercise without opposition, and thereafter 2 vs 2 small sided games (SSG) with floater was carried out. The final part of the session was 5 vs 5 or 6 vs 6 SSGs (dependent of the number of players) for 4^*^4 min, with 1.5 min recovery between games. See Appendix 1 for detailed information about the training session.

### Questionnaire and collection of questionnaire data

In the final training session each week, following two differentiated and two non-differentiated sessions, players completed a brief questionnaire assessing psychological variables such as perceived wellbeing, mastery, joy, satisfaction, development, and their preference for future sessions based on their experiences from the previous week. The psychological aspects being measured using a single item, selected for clarity and relevance to the training context. While single-item measures may have limitations in terms of psychometric depth, they are considered appropriate when the constructions are concrete, easily understood, and when participant burden must be minimized—particularly in youth populations. Fisher et al. (2016) showed that single-item measures can be appropriate when constructions are clearly defined, and when survey length or participant burden is a concern. Furthermore, the immediate post-session timing and repeated measures across conditions strengthen the reliability of the responses. The questionnaire was previously used in a study examining the effects of coaches' feedback on psychological outcomes in youth football (Berntzen and Lagestad, 2025), and comprised seven questions: “I have enjoyed the football training as it has been the past week,” “I have experienced mastery in the football training as it has been the past week,” “I have experienced joy in the football training as it has been the past week,” “I have been satisfied with the football training as it has been the past week,” “I feel I am developing as a football player with the football training as it has been the past week,” “I want to have the football training as it has been the past week,” and “I do not want to have the football training as it has been the past week.” Players responded using a Likert scale from 1-5 (Thomas et al., 2023), where 1 = strongly disagree, 2 = disagree, 3 = neither agree nor disagree, 4 = agree, and 5 = strongly agree. Five-point Likert scales are commonly used in questionnaire studies, offering five response options: two extremes, a middle (neutral) option, and two intermediate options. These scales are both valid and reliable for inclusion. A study by Østerås et al. (2008) demonstrated that the five-point scale ensures data quality, internal consistency, and discriminative validity, making it suitable for research. All questions (one measuring each variable) in the questionnaire had a high face validity—which is highlighted as important in studies (Holden, 2010). Furthermore, the questions were clear and concise—which we will argue is of importance among youth and had previously been used within youth football players (Berntzen and Lagestad, 2025).

### Measurement of physical data

To monitor and evaluate the physical variables such as total distance, number of sprints, number of accelerations, and average heart rate in the soccer players, a Polar Team Pro tracking system based on GPS technology was applied during all sessions. The Polar Team Pro system, which utilizes GPS and accelerometers, accurately measures distances and intensities in outdoor settings. A study by Akyildiz et al. (2020) confirmed the accuracy and interunit reliability of two Polar Team Pro units, deeming them suitable for tracking team sport variables. Similarly, Huggins et al. (2020) validated the reliability of Polar Team Pro units in measuring total distance and velocity in soccer athletes under outdoor conditions. They concluded that the Polar Team Pro units demonstrated good reliability during team sport movements outdoors. Players' movements were measured by a sensor located on the chest, which continuously monitored the physical variables. Total distance, number of sprints, number of accelerations, were registered at 10 Hz, whereas heart rate was sampled at 1 Hz. The selected locomotor categories for this investigation included total distance (all speed categories), acceleration zone 1 (Acc1) (2.00−2.99 m/s^2^) and acceleration zone 2 (Acc2) (>3.00 m/s^2^) were used. In line with previous research on youth players, the sprint threshold was set at 19 km·h^−1^, following the default manufacturer settings and consistent with earlier studies (e.g., Buchheit et al., 2010; Gualtieri et al., 2023). After training, the data were stored in a cloud-based server database for further offline processing.

### Data processing

Psychological data related to wellbeing, mastery, joy, satisfaction, and development in both differentiated and non-differentiated training sessions were entered into SPSS under each ID. Regarding the physical variables recorded by Polar Team Pro, the average for the two respective sessions with each organizational form was used for sprints, total distance, Acc1 and Acc2, and average heart rate for the entire session. Physical variables from the various exercises were extracted. The total distance was measured in meters, while the heart rate was measured as the average number of heart beats per minute.

### Data analysis

IBM SPSS Version 29 was used for statistical analyses (SPSS, Inc., Chicago, IL, USA). Kolmogorov-Smirnov tests showed violations of the normal distribution requirements of the variables (*p* < 0.05). To evaluate the effect of organizational differentiation related to skill level, the Wilcoxon non-parametric test was used to identify any differences between the differentiated and non-differentiated sessions, both in terms of psychological and physical variables (total training and different sub-exercises) (Thomas et al., 2023). We conducted analyses of total distance and average heart rate for the overall training session, the small-sided games (SSGs), and the 2vs2+1 SSGs. Data from the Rondo 4vs1 exercise were included only for heart rate analyses, as total distance is not considered a meaningful indicator of physical load in this specific drill. Analyses of sprint and acceleration variables were conducted for the total training sessions, comparing differentiated and non-differentiated training. Effect sizes were estimated using r (Z/√N), which provides a correlation-based measure of effect size for Wilcoxon tests, interpreted according to Cohen's conventional thresholds (0.1 = small, 0.3 = medium, 0.5 = large Cohen, 1988). Significant differences were set at *p* ≤ 0.05, and all data are presented as mean ± standard deviation to ensure consistency with previous studies and to enhance interpretability (Thomas et al., 2023).

## Results

### Differentiated trainings effect on psychological variables

**Figure 1:**
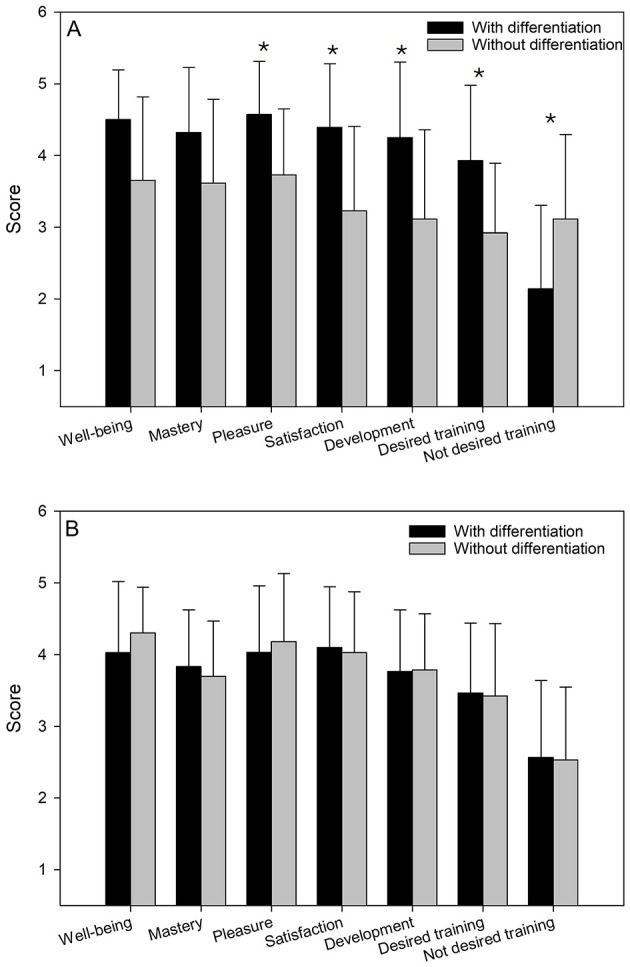
Experienced wellbeing, mastery, pleasure, satisfaction, (perceived) development, and desire for increased differentiated training sessions compared to non-differentiated training sessions among players in the upper-level group [UG, **(A)**] and lower-level group [LG, **(B)**]. Score in Y-axis: scale from 1 to 5, where 1 = strongly disagree, 5 = strongly agree. *Significantly different between differentiated vs. non-differentiated training (*p* < 0.05).

Analysis of players in the UG showed that according to wellbeing and mastery, there were no significant difference (*p* > 0.05) in the experience of wellbeing and mastery between differentiated and non-differentiated training sessions (Figure 1A). However, there was observed significant higher experience of pleasure (Z = −2.4, *p* = 0.015, r = 0.454), satisfaction (Z = 2.2, *p* = 0.025, r = 0.416), and perceived development (Z = 2.6, *p* = 0.008, r = 0.491) in differentiated training sessions compared to non-differentiated ones. Moreover, players in UG significantly preferred differentiated training sessions (Z = 2.7, *p* = 0.008, r = 0.510). Among players in the lower-level group (LG), there was no significant difference between differentiated and non-differentiated sessions (Figure 1B) in terms of experienced wellbeing, mastery, pleasure, satisfaction, and perceived development (*p* > 0.05). Furthermore, players in LG had no difference in the desire for differentiated training sessions compared to non-differentiated training sessions—even when controlled with a negatively worded question.

### Differentiated trainings effect on physical variables

**Figure 2:**
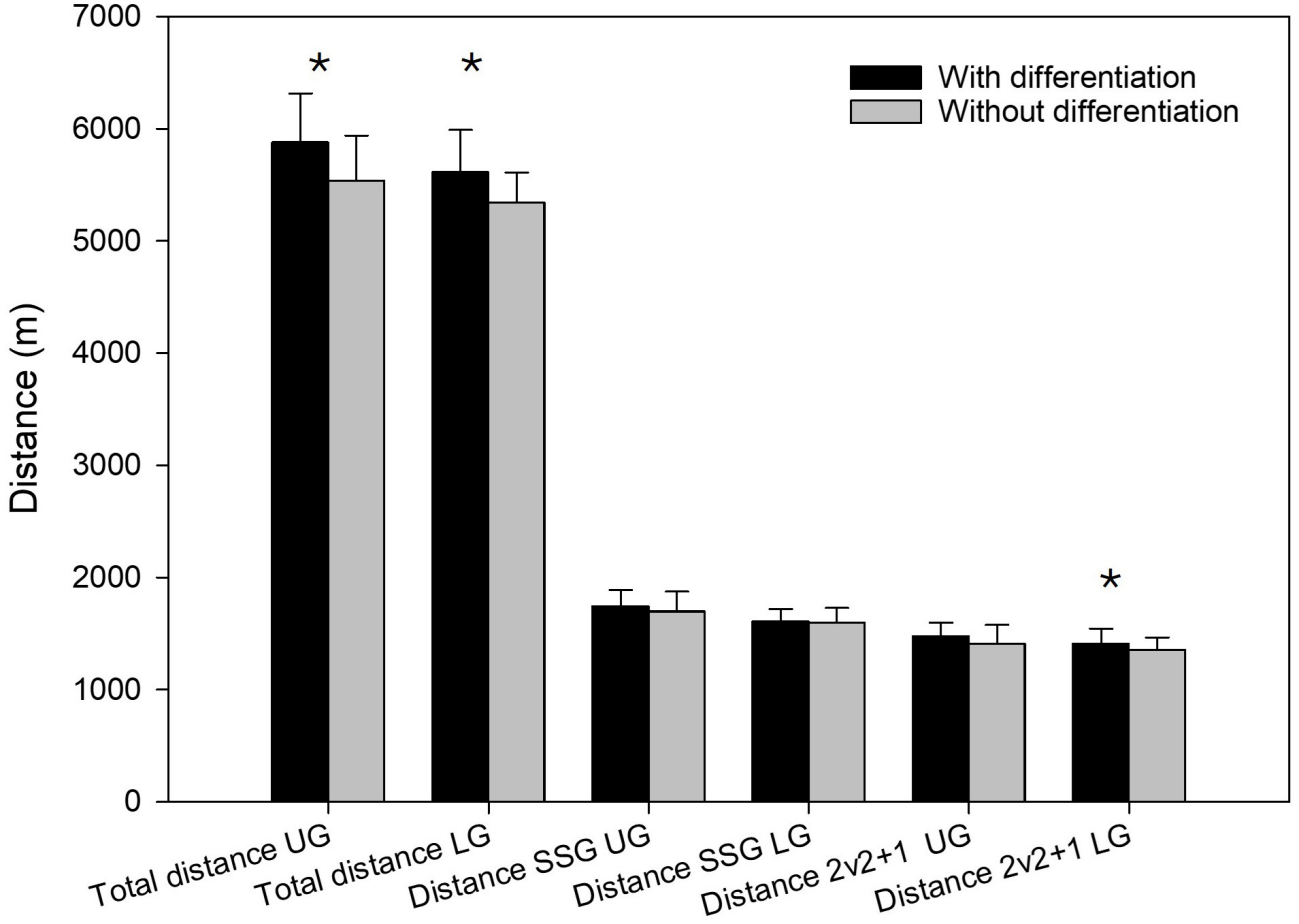
Total distance (TD), distance during small-sided games (SSG), and distance during 2v2+1 games for players with upper-level group (UG) and lower-level group (LG) in differentiated and non-differentiated training. *Significantly higher in differentiated vs. non-differentiated training (*p* < 0.05).

Players in both LG and UG registered a significantly higher total distance during the entire training in differentiated sessions compared to non-differentiated sessions (Z = −3.4, *p* = 0.003, r = 0.760 and Z = −2.7, *p* = 0.007, r = 0.493, respectively). However, no significant change was found in total distance between differentiated and non-differentiated sessions during small-sided games for both groups (*p* > 0.05). During the SSGs, there was no difference in total distance for players in UG, while there was a higher total distance SSGs (2vs2+1) in differentiated training compared to non-differentiated training for players in LG (Z = 2.3, *p* = 0.023, r = 0.325). See Figure 2 for more information.

**Figure 3:**
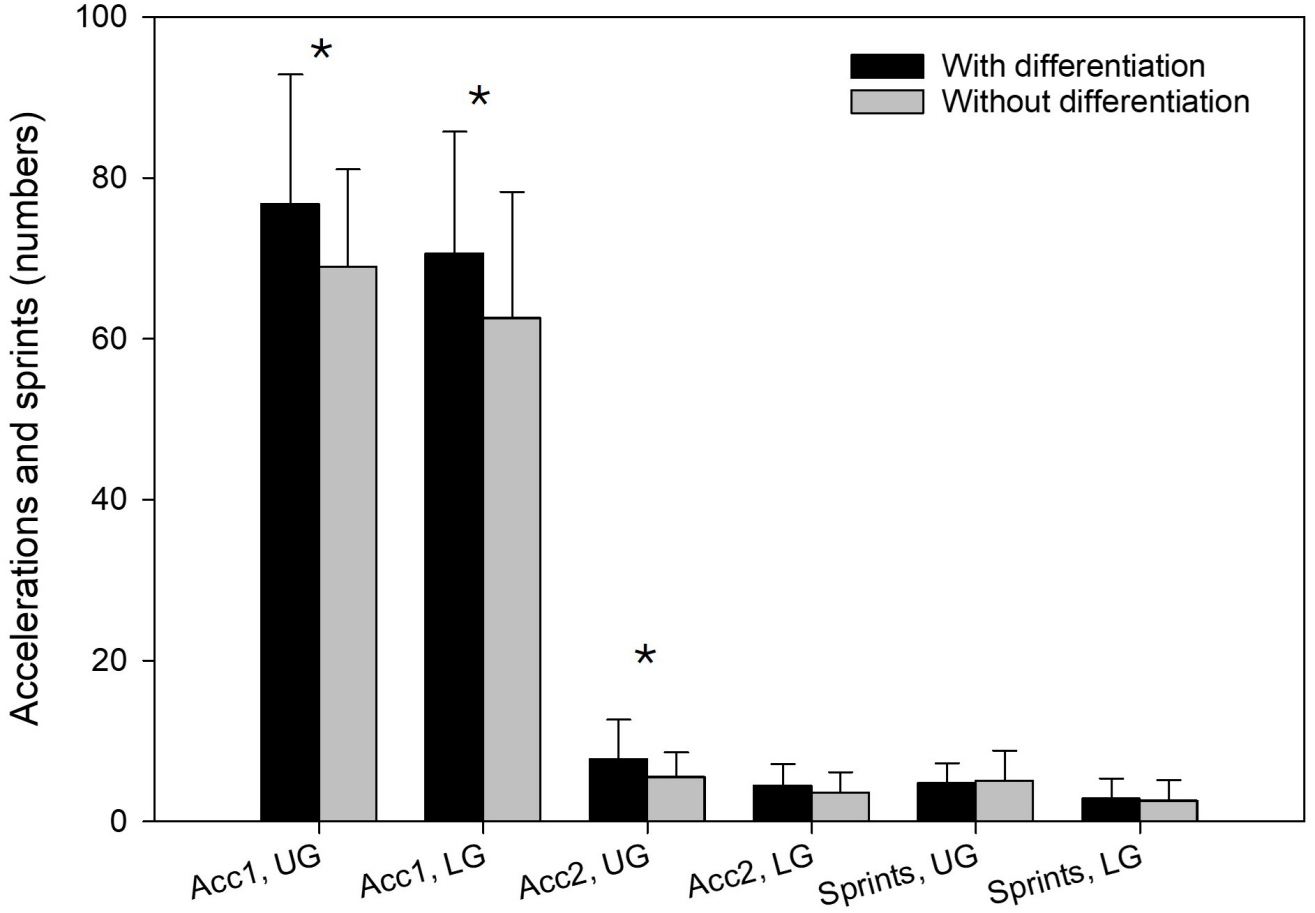
Number of accelerations and sprints for players in upper-level group (UG) and lower-level group (LG) during the entire training session in differentiated and non-differentiated training. *Significantly higher in differentiated vs. non-differentiated training (*p* < 0.05). UG, upper-level group; LG, lower-level group; Acc1, acceleration zone 1; Acc2, acceleration zone 2.

Figure 3 presents accelerations and sprints among players in UG and LG in differentiated and non-differentiated sessions. Players in both UG and LG showed significantly higher number of accelerations between 2 and 3m/s^2^ during differentiated training compared to non-differentiated training (Z = 2.1, *p* = 0.032, r = 0.383 and Z = −2.4, *p* = 0.016, r = 0.587, respectively). However, none of the groups showed a change in the number of sprints between differentiated and non-differentiated training in the SSGs (*p* > 0.05). For accelerations above 3m/s^2^, there was no significant difference for players in LG during differentiated training compared to non-differentiated training (*p* > 0.05), while there were significantly more accelerations during differentiated training compared to non-differentiated training for players in UG (Z = −2.5, *p* = 0.011, r = 0.456).

**Figure 4:**
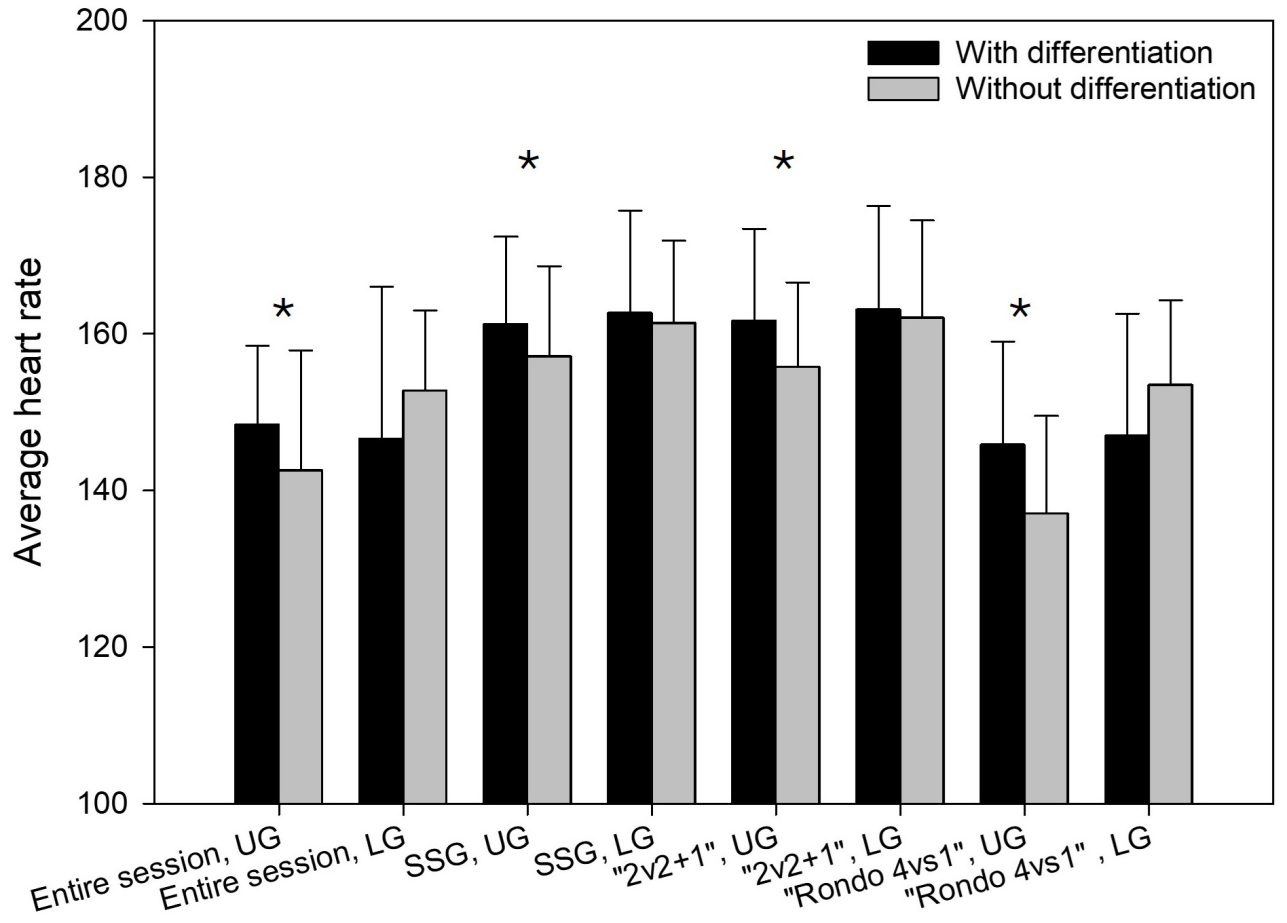
Average heart rate for the entire session, SSGs 2v2+1 games, and rondo (4v1) among players in upper-level group (UG) and lower-level group (LG) during differentiated and non-differentiated training sessions. *Significantly higher in differentiated vs. non-differentiated training (*p* < 0.05).

Figure 4 presents the average heart rate among players in UG and LG, in differentiated sessions and non-differentiated sessions. The analysis show that UG had a significantly higher heart rate during differentiated training compared to non-differentiated sessions throughout the training (Z = 2.6, *p* = 0.010, r = 0.475), but also during SSGs (Z = 2.2, *p* = 0.026, d = 0.382), 2v2+1 games (Z = −3, *p* = 0.003, r = 0.402), and in the exercise Rondo 4vs1 (Z = 3.4, *p* < 0.001, r = 0.621). However, for the group with below average skills, there were no significant differences in heart rate in differentiated training compared to non-differentiated training, neither for the entire training nor for parts of the training (*p* > 0.05).

## Discussion

The study found that players in the upper group (UG) experienced significantly higher enjoyment, satisfaction, and development during differentiated training compared to non-differentiated training, unlike players in the lower group (LG). UG players also preferred differentiated training, while no preference was observed in LG. Regardless of group, all players had more accelerations and covered greater total distances during differentiated training. Additionally, UG players exhibited a higher average heart rate throughout the session and in specific sub-exercises such as SSGs, 2v2+1 games, and rondo 4v1. The findings are in line with the study hypothesis that organizational differentiation in high and low skill level will have a positive effect on the physical variables, but regarding psychological variables such differentiation only influenced the UG significantly.

The current study highlights the effect of differentiated sessions compared to no-differentiated sessions in UG and LG separately in terms of enjoyment, satisfaction, and development. Creating enjoyment and satisfaction is crucial to prevent dropouts in sports, and differentiation can be an important tool in this regard. Seipel (2005) found that 20% of youth players dropped out when they no longer found it enjoyable. According to Eime et al. (2013), players reporting more development and satisfaction with differentiated training is important, as the experience of development is a key factor in creating enjoyment in sports and positively impacts self-esteem. The Norwegian Sports Federation's policy document states that “sports enjoyment for all” is a central goal. This is echoed by NFF, which emphasizes that “the main goal of children's and youth football is to provide children and young people with good football opportunities and experiences” (NFF, 2021b). The NFF's vision for football includes “As many as possible, as long as possible, as good as possible,” and “Football for all—Joy, dreams, and community.” The current study's finding regarding perceived enjoyment, satisfaction, and development has shown that differentiation can positively influence this goal compared to non-differentiated training. The increased development experienced by players is attributed to the adaptation of training to different skill levels (Berntsen and Johansen, 2020). NFF also emphasizes differentiation as a key tool to achieve these goals (NFF, 2021a). This aligns with Bon's (2009) research, which highlights that differentiation can foster more favorable development, particularly for those with the greatest potential. However, Bon (2009) and the Norwegian Directorate for Education and Training (2023) stress the importance of maintaining social belonging. Extensive differentiation over time may negatively impact young people by limiting their interactions with different teammates.

The fact that UG players preferred differentiated training, while no preference was observed in LG could indicate that differentiation in training facilitates individualization. Allowing players to experience individualization in sports is an important tool for influencing development and enjoyment among players (Hallèn and Ronglan, 2022). Hallèn and Ronglan highlight the importance of considering the various personal and athletic prerequisites to achieve individualization in a sports group. Some evidence suggests that for those with above-average skills, differentiation makes the training more tailored to the individual player (Hallèn and Ronglan, 2022). Lower group players did not experience any significant difference in perceived wellbeing, mastery, joy, satisfaction, and development in differentiated and non-differentiated training sessions may suggest that the players are there for reasons other than becoming the best possible in football, and that participation is based on social reasons. Considering Berntsen and Johansen's (2020) formula “wellbeing = mastery x security,” this suggests that they experience as much mastery and security in differentiated as in non-differentiated training sessions. The findings indicate that wellbeing is high regardless of the organizational form, suggesting that wellbeing can be achieved in both differentiated and non-differentiated sessions. Johansen (2019) emphasizes the importance of supporting all players, regardless of skill level. Differentiation in training does not negatively impact psychological and physiological variables, as players experienced similar levels of joy in both training types. This is positive, as players thrive regardless of their opponents. The fact that players experience joy regardless of their opponents is beneficial, considering the potential emotional impact (Bon, 2009). However, while our findings support the positive impact of organizational differentiation on physical and psychological outcomes for UG players, it is important to acknowledge potential negative consequences for LG players. Although our data did not show a significant decline in psychological or physical outcomes for LG players due to differentiation, the absence of preference for differentiated training in this group may suggest a more neutral or even ambivalent experience. Previous research has shown that differentiation based on cognitive performance may unintentionally contribute to stigmatization and reduced self-esteem among lower-skilled students, particularly when grouping is perceived as a reflection of ability or potential (Haelermans, 2022) emphasizes that such practices can lead to social exclusion and diminished motivation, especially if not accompanied by inclusive pedagogical strategies and teacher awareness.

In the current study, all players exhibited more accelerations and covered greater distances during differentiated training compared to non-differentiated training, regardless of group. The ability to make quick accelerations during a football match is crucial (Mohr and Bangsbo, 2005). In order to illustrate the substantial physical and locomotor demands placed on top-level players during competitive match play, Mohr and Bangsbo (2005) reported that international elite soccer players typically perform around 1,350 repeated movements during a match, including approximately 220 actions at high speed. Thus, the players' ability to make quick accelerations is an important measure in relation to the demands of football. Given the importance of individualization, the increase in accelerations supports the argument for differentiated training. This approach aligns with Bolotin and Bakayev (2017), who ranked differentiation and individualization as the second most important pedagogical tools for enhancing speed and explosive strength in younger football players. Evidence suggests that differentiation ensures players receive appropriate challenges and push themselves more, which is crucial for development (Johansen, 2019). Developing physical capacity through accelerations is vital for football success (Skjerping, 2020) and must start early. This increases tolerance for high training loads (Bompa and Haff, 2009), enhancing the development of football skills. Field size is a key factor for achieving many accelerations; smaller spaces, as shown in the session plan, require frequent quick movements (Santos et al., 2021). Thus, differentiation aligns with the principle of individualization, promoting more training on accelerations.

Increased total distance suggests a higher training load, indicating that players can tolerate more training and have the potential for increased training volume. The results from UG indicate that players run more when they are differentiated. Considering total distance during a session as a measure of physical load, the focus is primarily on aerobic capacity. The significant increase in running during differentiated sessions compared to non-differentiated sessions is crucial for physical development. Running more each session will enhance aerobic capacity over time, helping players meet the match requirement (Mohr and Bangsbo, 2005).

The current study found that players in UG had a higher average heart rate during the entire session, as well as in the sub-exercises SSG, 2v2+1 games, and rondo 4v1. Previous studies have shown that SSGs have the same positive effect on endurance performance as traditional endurance training (Reilly and White, 2004). The results of this study suggest that differentiated sessions may be more effective than non-differentiated small-sided games for endurance development. The increase in average heart rate during differentiated training indicates higher intensity in physical conditioning, leading to better development if recovery is adequate (Hallèn and Ronglan, 2022). Ronglan (2018) emphasizes the importance of adapting the training to the players' abilities, which differentiated training better facilitates. High-intensity aerobic endurance develops when the average heart rate is between 87–97% of the maximum (Bompa and Haff, 2009). This indicates that a higher average heart rate influences the capacities developed. In elite junior players, the average intensity is close to the lactate threshold, at 87–90% of maximum heart rate (Hoff and Helgerud, 2004). Therefore, it is crucial for players to train at high intensity during the week, which differentiation better facilitates compared to non-differentiation. Hoff and Helgerud also found that training at 90–95% of maximum heart rate had a greater effect on aerobic endurance than training at 60–80%. Thus, a higher average heart rate has a greater impact on the aerobic endurance of football players than lower intensity training.

### Discussion of main findings considering flow theory

Flow theory suggests that balancing challenge and skills positively influences intrinsic motivation (Csikszentmihaly, 1975, 1990; Hood, 2007). The positive effects of differentiation can be explained by this theory, as optimal challenges are crucial for achieving flow (Biasutti, 2011). The results might indicate that players in UG most frequently entered the flow state during differentiated training sessions (Csikszentmihaly, 1975, 1990), experiencing the most joy, satisfaction, and development. When players enter the flow state, their focus is highest (Biasutti, 2011) due to appropriate challenges. Enjoyment is also highest during differentiated training, leading to more enjoyment around hard training (Csikszentmihaly, 1975). Both UG and LG achieved longer distances and had significantly more accelerations (2–3 m/s^2^) during differentiated training compared to non-differentiated training. These findings align with flow theory, which explains the best sports experiences as being in the flow state, where skills and challenges match (Csikszentmihaly, 1990). Players' ability to recognize and create challenges within the training framework is central to experiencing the flow state (Hood, 2007). When players do not experience the flow state, it may be because they cannot recognize the challenge and use their skills while enjoying the activity (Hood, 2007). Csikszentmihaly (1990) identifies eight dimensions of enjoyment and pleasure, where certain criteria are important for athletes to experience the flow state. One criterion is to give players clear goals and defined tasks, which can lead to players feeling that their skills match the challenge, that their concentration is related to the task, and that they have potential control (Csikszentmihaly, 1990). To achieve this, the coach's competence in providing the right challenges is crucial for players to enter the flow state.

Biasutti's (2011) research has shown that the flow state arises from intense activities, either through intense focus or physically intense training. For players in UG, higher intensity during differentiated sessions compared to non-differentiated sessions is evident. The significantly higher average heart rates, total distances, and accelerations in UG can be explained by the flow theory. As Csikszentmihaly (1990) describes, players become absorbed in the activity when they enter the flow state. Thus, intrinsic motivation and self-confidence increase (Hektner and Csikszentmihalyi, 1996), leading to longer and faster running and higher average heart rates. In differentiated training, players with above-average skills face greater challenges, requiring more effort to succeed. This increased effort leads to higher intensity, supported by the results. In UG, moving more and faster is essential for success, explaining why total distance, heart rate, and accelerations were higher in differentiated sessions compared to non-differentiated sessions.

### Strengths and weaknesses of the study

A strength of this study is that it is the first to investigate how psychological and physical factors are affected by differentiation. The use of an experimental crossover design, measuring differentiated and non-differentiated training through two sessions each, on the same fields, with the same exercises, at the same time, and with the same coach, enhances the credibility of the findings. Athlete responses to questionnaires were collected immediately after the last training session each week, and we argue that the questions have high immediate validity and use validated Likert scales (Thomas et al., 2023; Østerås et al., 2008). Although the questionnaire was not pre-validated, we argue that the questions and reply options have high face validity (Holden, 2010), reducing the likelihood of varied interpretations and increasing reliability. Another strength is the collection of objective data through Polar Team Pro for physiological data. However, the study has several weaknesses. A limitation of this study is that potential effects of season timing were not controlled for. However, as both groups were exposed to differentiated and non-differentiated training independent of the time of year, we consider this risk to be minimal. More participants could have been included, and although the questions used have high immediate validity, more questions could have been used to measure each attribute. Although no wash-out period was used between conditions, the short-term nature of the intervention and immediate post-session data collection reduce the likelihood of carryover effects. Nonetheless, the absence of a wash-out period may be considered a limitation. One limitation of the present study is the absence of a priori power analysis to determine the required sample size. While the sample size was based on practical constraints, this may affect the precision and interpretability of the statistical conclusions. Finally, the grouping of players into upper-level (UG) and lower-level (LG) was determined solely by the coaches' subjective evaluations. Although this approach may introduce selection bias and limit reproducibility, it reflects the reality of player assessment in practice, where coaches are both the primary evaluators and the individuals most qualified to judge their athletes.

## Conclusion

This study demonstrates that organizational differentiation in youth football training can enhance physical outcomes for all players and psychological outcomes particularly for higher-skilled players. Upper-level players experienced greater pleasure, satisfaction, and perceived development in differentiated sessions, while lower-level players did not report significant psychological changes but still benefited from increased physical load. Importantly, no negative short-term effects were observed.

In practice, differentiation can be a useful strategy for coaches to balance challenges and skill, thereby increasing training intensity and fostering positive experiences, and football coaches should organize their training with differentiation according to the players skill level. However, careful consideration should be given to the potential long-term consequences for lower-skilled players, especially regarding social belonging and self-perception. Future research should explore these aspects in greater depth and examine the longitudinal effects of differentiation in youth sport.

## Data Availability

The raw data supporting the conclusions of this article will be made available by the authors, without undue reservation.
