# Supplementary material for: The effect of organizational differentiation in football training on young football players
Source: Front Psychol. 2025 Nov 11;16:1565594. doi: 10.3389/fpsyg.2025.1565594 (PMC12643876; doi:10.3389/fpsyg.2025.1565594)
Supplement: Supplementary file 1 [file Table_1.docx]

Appendix 1

Appendix 1: Organization of the Training Session

| Time | What | How |
| --- | --- | --- |
| 5 min | General Warm-up | «Injuri-free» (by NFF) |
| 10 min | Specific Warm-up | 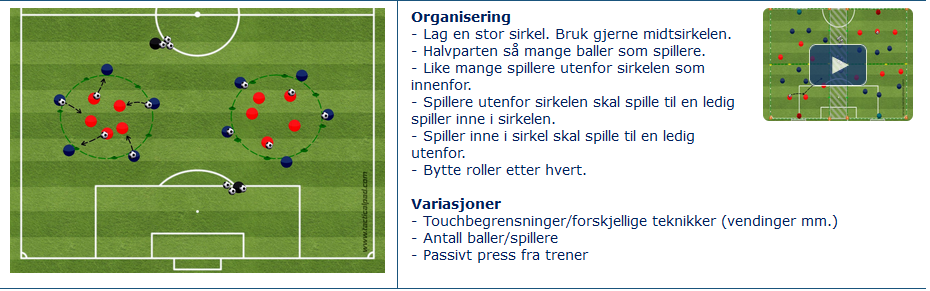  **Organization**   - Create a large circle. Preferably use the center circle. - Half as many balls as players. - Equal number of players outside the circle as inside. - Players outside the circle should pass to an open player inside the circle. - Players inside the circle should pass to an open player outside. - Switch roles after 45 seconds.   **Variations**   - Two touches - One touch - Chest control and pass back - Turn and pass to a new person outside the circle |
| 10 min | Technical exercise. «Dutch» | 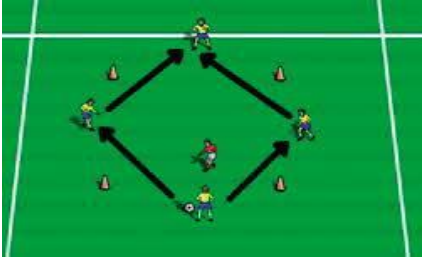  **Organization**   - Four people outside - If the person in the middle intercepts the pass, they switch with the person who made the pass   **Variations:**   - Free touch - 2 touches - 1 touch   3 minutes and 20 seconds for each variation. |
| 10min | Passing exercise | 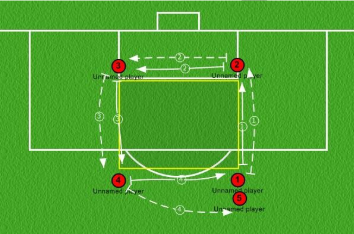  **Organization**   - Pass to a player who comes to meet the ball. The player who meets the ball takes a touch past the back of the cone. Then it is passed on to cone number three. They move after the ball is passed.   **Variations**   - Same as variant 1, but now with a wall pass. - Both exercises are performed for a total of 5 minutes, but we change direction halfway through. |
| 20 min | Outnumber  Game | 2v2+1. Small-sided game with a joker.  Field size: 10x20 meters  5x3 minutes. 1-minute break between games  Score by dribbling the ball over the opponent’s line |
| 25 min | SSG | 5v5 or 6v6  Field size: 40x20 meters  4x4 minutes. 1.5-minute breaks |

The training session was organized as a typical and traditional football training for this age group based on the recommendations of the Norwegian Football Federation, and the same coach conducted all the training sessions (Appendix 1). The exercises were developed based on traditional football training sessions. The teams started the training by performing part of the warm-up exercise “FIFA 11+” followed by a technical circle for a more specific warm- up. After that the players performed a rondo 4vs1-exercise in squares of 5x5 meters. The rondo exercise was followed by a passing exercise without opposition, and thereafter a 2 vs 2 small sided game (SSG) with floater was carried out. The final part of the session was 5 vs 5 or 6 vs 6 SSGs (dependent of the number of players) for 4*4 mins, with 1.5 mins recovery between games. See Appendix 1 for detailed information about the training session. In the passing exercise, the size of the squares was 10x10 meters. Before each exercise, it was demonstrated how the players should perform it, and 5 minutes were spent on both variants. After 2.5 minutes, the direction of the passes was changed. During the game part with 2 versus 2 + 1, there were some variations in group size, sometimes 3 versus 3, but most often 2v2 + 1. The joker was changed every third minute to make it as fair as possible for everyone. Between the work periods of three minutes, the players had a one-minute break. The last part with small-sided games (40x20 meter field) had games lasting four minutes, with 1.5-minute breaks between game sessions, with team sizes ranging from 4 to 7 players, but most often 5 versus 5 or 6 versus 6 depending on the number of players available. The shortest training session lasted 1 hour and 33 minutes, while the longest took 1 hour and 45 minutes. The reason for this time difference was related to the organization of new activities and limited field area. Most sessions lasted 1 hour and 38 minutes. The same coach performed every training sessions.
